# Parent and healthcare professional experiences of critical congenital heart disease in New Zealand to advance health equity

**DOI:** 10.1186/s12913-024-11410-4

**Published:** 2024-08-26

**Authors:** Simone Watkins, Kim Ward, Rachel Brown, Sue Crengle, Monique WM de Laat, Teuila Percival, Lynn Sadler, Elza Cloete, Ruth Gorinski, Thomas Gentles, Frank H. Bloomfield

**Affiliations:** 1 Liggins Institute, University of Auckland, Auckland, New Zealand; 2 School of Nursing, University of Auckland, Auckland, New Zealand; 3 National Hauora Coalition, Auckland, New Zealand; 4 Ngāi Tahi Māori Health Research Unit, Division of Health Sciences, University of Otago, Dunedin, New Zealand; 5 Te Toka Tumai (Auckland hospital), Te Whatu Ora, Auckland, New Zealand; 6 Department of Paediatrics: Child and Youth Health, University of Auckland, Auckland, New Zealand; 7 Te Whatu Ora (Christchurch hospital), Christchurch, New Zealand; 8 Heart Kids NZ, Tamariki Manawa Māia, Auckland, New Zealand

**Keywords:** Congenital heart disease, Equity, Indigenous, Qualitative research

## Abstract

**Background:**

Higher odds of survival have been reported in European infants compared to Indigenous Māori and Pasifika infants with critical congenital heart disease in New Zealand. We therefore aimed to understand how to mitigate this disparity by investigating the parent and healthcare professional experiences’ of critical congenital heart disease healthcare in New Zealand.

**Methods:**

A prospective qualitative study utilising semi-structured interviews was conducted on a cohort of purposefully sampled parents and health professionals with experience of critical congenital heart disease healthcare in New Zealand. Parents were recruited after a fetal critical congenital heart disease diagnosis and offered two interviews at least three months apart, whilst multidisciplinary fetal and cardiosurgical health professionals were interviewed once. Interviews were recorded and transcribed verbatim before coding, categorization and qualitative analysis.

**Results:**

During 2022 and 2023, 45 people participated in 57 interviews (25 parents: 19 mothers, 6 fathers; Indigenous Māori, *n* = 5; Pasifika, *n* = 6; Asian, *n* = 4; European, *n* = 10; and 20 healthcare professionals: European *n* = 17). The three lessons learned from participants were: (1) Minoritized groups experience disparate healthcare quality; (2) healthcare systems are under-resourced to provide equitable support for the differential needs of grieving parents; and (3) healthcare systems could engage minoritized families more optimally in shared decision-making.

**Conclusions:**

According to the experiences of parents and healthcare professionals, persisting inequities in CCHD healthcare quality occur by ethnic group, with the New Zealand healthcare system privileging European families. The concepts from this study could be translated by healthcare leaders, policymakers, and professionals into evidence-based healthcare system improvements to enhance experiences for non-European families more broadly.

**Supplementary Information:**

The online version contains supplementary material available at 10.1186/s12913-024-11410-4.

## Background

Congenital heart disease (CHD) is a leading cause of infant mortality [1]. Parents and healthcare professionals caring for CHD children report psychological challenges, particularly with complex life-limiting critical CHD (CCHD) conditions such as hypoplastic left heart syndrome (HLHS). [2–4] Further complicating CHD care are the differences in understanding between multidisciplinary health professionals and parents [5–7]. 

Over time, the modernization and sophistication of diagnostic and treatment methods in CCHD have significantly improved life expectancy [8]. However, disparate CCHD survival by ethnicity and race persist, with minoritized racial groups (Indigenous, Blacks and people of colour) most negatively impacted [9]. Even in New Zealand (NZ), a universal, tax-funded healthcare setting, disparate management pathways and infant survival are reported, with superior HLHS outcomes for European (NZ European, other European, “White”) compared to Indigenous Māori and Pasifika infants [10, 11]. 

Moreover, patients from minoritized ethnic groups are historically under-represented in quantitative research generally, with specific paucity in the documented CCHD healthcare experience of minoritized ethnicities and healthcare professionals to inform equity improvements [12, 13]. Since quantitative approaches gather limited insights into why inequities in health outcomes arise due to the nuances of complex human-centered healthcare issues, [14, 15] we undertook a qualitative study to enrich knowledge in healthcare professional and parent experiences of CCHD to identify possible contributors to ethnic disparity in infant outcomes.

## Methods

### Study design

A qualitative study using grounded theory methods and semi-structured interviews was conducted in NZ from June 2022 to April 2023 (Supplementary Tables 2–4: Semi-structured interview guides) [16]. Grounded theory allowed a flexible but systematic approach to generating categories grounded in the data to develop a theory to guide action [14]. To ensure that Indigenous and minoritized perspectives were shared, Kaupapa Māori [17] and Talanoa [18, 19] principles were interwoven into a culturally-sensitive approach appropriate for the NZ setting (Supplementary Table 1). Māori developed Kaupapa Māori research theory to validate their worldview (cultural approaches, values and beliefs) [17, 20] and to overcome deficit narratives within Western research approaches [17, 20]. Kaupapa Māori has been defined as:

“having a collective vision [which] binds people to one another for the achievement of [Māori] cultural wellbeing and prosperity” [21].

Similarly, Pasifika people developed specific culturally-conducive guiding research principles for their people, focusing on relationship, partnership, and community within the Talanoa methodology [18, 19]. Talanoa values reciprocity, respect, and open dialogue [22]. Compared to Western methodologies, Talanoa focuses on oral knowledge production and sharing of information through conversation that is usually face-to-face (Supplementary Table 1) [22]. 

### Participants

Prospective recruitment of parents and healthcare professionals occurred in NZ’s sole paediatric cardiac surgery centre (Starship Child Health), all three maternal fetal medicine (MFM) centres within NZ (Auckland, Wellington and Christchurch hospitals) and the Auckland MFM hub in South Auckland (Counties Manukau). Parents with a fetal diagnosis of CCHD, irrespective of additional comorbidities, evolving cardiac diagnoses and potential outcome (live birth, stillbirth or termination) were eligible for inclusion, with no pre-specified exclusion criteria. Cases were identified by the MFM teams and communicated to the lead author. An invitation to parents was given via flier, phone call and email. Purposeful recruitment (where individuals were sampled to inform developing categories) occurred until theoretical data saturation. Health professionals, including MFM specialists, pediatric cardiologists and cardiothoracic surgeons, fetal medicine midwives, and pediatric cardiology specialist nurses who worked closely with CCHD families, were invited via a personal email invitation. All participants were offered a koha [gift] of $50 per interview for their time.

### Ethics and funding

Ethics approval was granted by the NZ Health and Disability Ethics Committee on 04/07/2021 (reference number: 21/CEN/128), and additional locality approval was obtained from each participating hospital. All participants were informed (in their own language if applicable) about the research purpose and process, and of their rights, and were assured of confidentiality and anonymity. Informed consent to participate was obtained from all of the participants in the study. The parent participants had no relationship with, or knowledge of, the interviewers before the study. The study funder, the Health Research Council of New Zealand, had no role in the study beyond funding.

### Transparency statement

The lead author affirms that the manuscript is an honest, accurate, and transparent account of the study being reported and that no important aspects of the study have been omitted.

### Data collection

Interviews were conducted in person or via audio or video call, and recorded and transcribed verbatim, before analysis. Interviews were conducted primarily by the first author (SW, female, Pasifika/European ethnicity, medical doctor, doctoral candidate, *n* = 50) with support from a Māori interviewer (RB, female, Te Ātiawa, Kāi Tahu, post-doctoral researcher, *n* = 7) for the Māori interviews; both had previous qualitative interview experience. One interview was with an interpreter and only the English was transcribed. Two semi-structured in-depth interviews were offered to parents. Interview questions evolved during analysis (copies of all versions of interview questions available on request). The first parent interview was offered after CCHD fetal diagnosis (allowing at least two weeks after the initial diagnosis was confirmed), and the second interview was offered at least three months later. All interviews were completed within 12 months of diagnosis. A single one-on-one interview was provided to healthcare professionals with static semi-structured interview questions with SW, a previously known colleague of some participants. Demographic questions were asked within the interviews. Field notes were kept throughout. Recruitment ceased once theoretical data saturation was reached.

### Data analysis

Data were stored and managed using NVivo software and Microsoft Word and analysed using grounded theory methods. Transcripts were offered for member checking. Analysis of the transcripts (SW) occurred through reading, re-reading, coding, re-coding, categorizing, re-categorizing, memo-writing, constant comparison, and diagramming, which was an iterative process [14, 15]. Two interviews were observed, and one transcript separately coded, by KW. Key findings were constantly cross-checked with KW during the research process and with close cultural oversight. Developing categories were presented to the co-authorship group for discussion and deliberation. Rigor was maintained by adhering to the consolidated criteria for reporting qualitative studies checklist (Supplementary Table 5) [23]. Credibility was sustained through member-checking, two interviews offered with parents, and triangulation to corroborate results (through interviews with the healthcare professionals). The writing of notes and memos during the process of analysis supported author reflexivity, and regular discussion and debate with co-authors, who had access to, and regularly reviewed, codes and quotes refined development of the theoretical concepts and, enhanced rigor. Where there was disagreement between co-authors, interview transcripts were re-reviewed and then re-discussed, to ensure results accurately reflected the data. Deidentified raw data are available from the authors upon request and review by the institutional data access committee.

### Patient and public involvement

The patients were involved in the study during the data collection phase, and the results are directly based on their interview storylines. There was no patient or public involvement in the research priorities or study design, recruitment, or dissemination strategy.

## Results

**Fig. 1:**
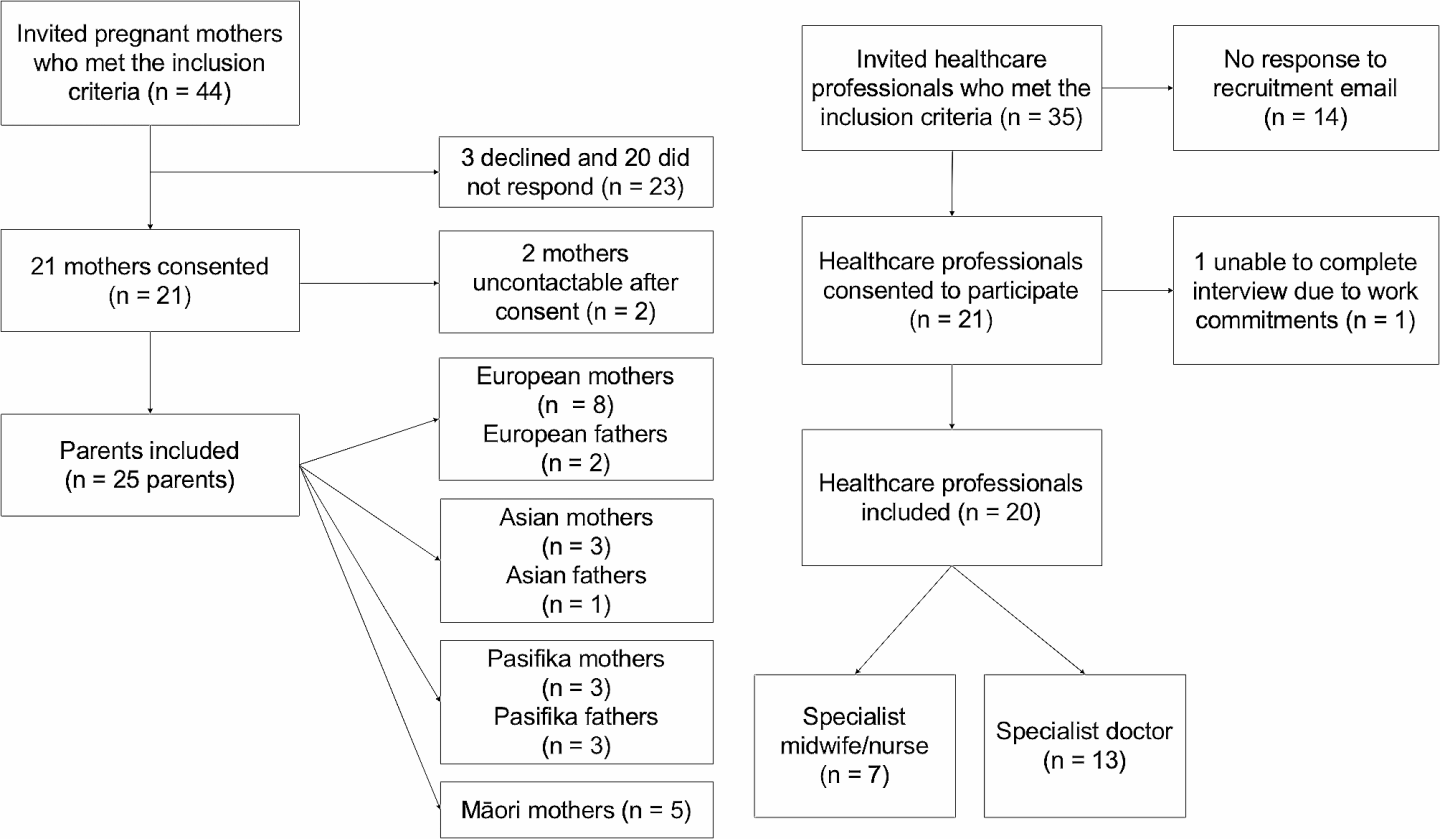
Recruitment process for parents and healthcare professionals

A total of 44 mothers and 35 healthcare professionals were invited to participate (Fig. 1). Of these, 25 parents and 20 health professionals (*n* = 45) partook in 57 interviews (Table 1) in which data codes within supplementary Fig. 1 were identified. No participant requested their data be deleted or withdrawn from the study and the majority did not change their transcripts. There were 19 mothers’ and 4 fathers’ experiences in the first interview and 18 mothers’ and 3 fathers’ experiences in the second interview. During the follow-up interviews, two fathers were recruited into the final participant count as part of the theoretical sampling process to increase the representation of fathers’ experiences.

**Table 1:** Parent and healthcare provider characteristics and interview information

Characteristics	Parents (*n* = 25)	Healthcare professionals (*n* = 20)
Sex	Female (identified as the mother) (*n* = 19) Male (identified as the father) (*n* = 6)	Female (*n* = 17) Male (*n* = 3)
European* [% experienced palliative care]	(*n* = 10) [10%]	(*n* = 17)
Non-European^ [% experienced palliative care]	Māori (*n* = 5) [40%] Pasifika (*n* = 6) [67%] Asian (*n* = 4) [50%]	(*n* = 3)
Location	Auckland (*n* = 15) Outside of Auckland (*n* = 10)	Auckland (*n* = 16) Outside of Auckland (*n* = 4)
Outcome+ (*n* = 21)	Termination of pregnancy (*n* = 2) Neonatal/infant all-cause death (*n* = 7) Alive (*n* = 12)	N/A
Average interview time (range)	*43 min (range of 5–113 min)*	*61 min (range of 40–107 min)*
Mode of interviews	*Phone call (n = 11)* *Video-call (n = 13)* *In-person (n = 13)*	*Phone call (n = 2)* *Video-call (n = 13)* *In-person (n = 5)*

* Ethnicity as documented within the maternal medical records or self-identified in the interview: one Māori mother also identified as Pasifika but is listed as Māori for ease of reporting

^ The non-European healthcare professionals specified their ethnicity, but we do not report on these data due to the high risk of identifying these participants

+ Two twin pregnancies were included, of which one twin was diagnosed with CCHD

The three categories contributing to inequity in CCHD outcomes by ethnicity in NZ identified by participants were: (1) Minoritized groups experience disparate healthcare quality; (2) healthcare systems are under-resourced to provide equitable support for the differential needs of grieving parents, and (3) healthcare systems could engage minoritized families more optimally in shared decision making (Supplementary materials, Table 6: Categories, subcategories and quotes).

### Category one: Minoritized groups experience disparate healthcare quality

Receiving discriminatory, poor-quality healthcare was experienced by some minoritized parents (Māori, Pacific, Asian, poor mental health, rural, living in deprivation). Identified contributors to poor healthcare quality included information-withholding, stereotyping, assuming, poor communication, and misunderstood cultural values. Several healthcare providers also described concerns that the healthcare system they worked within was ill-adapted to deliver healthcare to other minoritized groups such as parents belonging to the LGBTQ+ (lesbian, gay, bisexual, transgender, queer, and/or questioning) community, parents using surrogates, and pregnant people under the age of 16 years.

Comparatively, a high-quality healthcare services fit for purpose were most prominently voiced by European parents, stating their gratitude and lack of discrimination in quotes such as:

*“We were aware that we’re really fortunate in New Zealand to have such a level of [health]care.”* - European mother (P9).

*“We were blown away by how good everyone was.”* - European father (P25).

*“I’m pretty privileged that [discrimination is] not something that we’d normally experience.”* – European mother (P3).

Correspondingly, a discordant translation of equity ideals described by healthcare professionals transpired, with one doctor (HCP2) acknowledging “we’ve always just tried to be fair to everyone”. For example, parents of Indigenous Māori ethnicity reported the effects of stereotyping and subconscious racial biases on healthcare quality despite healthcare professionals’ best intentions.

*“My experiences with my partner*,* he’s Māori and you know*,* we [as parents] had social workers come up to the hospital when he [their son] was born*,* and they pretty much accused the reasoning behind me going into labour ten weeks early was because I was in a violent relationship*,* you know*,* and it’s just unnecessary. And I honestly believe it was because he’s quite rough-looking*,* you know… Don’t make assumptions because you don’t know.”* - Māori mother (P21).

*“To be honest*,* I think that they [the healthcare professionals] would go the extra mile for — well*,* it’s pretty sad to say*,* but they [the healthcare professionals] would go the extra mile for Pākehā [Europeans].”* - Māori mother (P2).

In keeping with the above, one healthcare provider explained the challenges involved in serving Māori families:

*“I think it is harder to gain full rapport with particularly some of the different ethnic group women*,* and that’s obviously a limitation for all of our healthcare. If we spoke te reo*,* [the Indigenous Māori language] we obviously try and learn a little bit*,* but it’s hard to — yeah.”* – Doctor (HCP2).

An essential element of healthcare quality was the level of health professional communication. Some minoritized parents felt unheard and experienced inconsistencies in care, usually within culturally discordant patient-provider interactions. For instance:

*“Every time [the medical team offer termination] and*,* I said*,* ‘No.’ To me with us*,* we know the Bible really well*,* and we are Islanders*,* so we don’t do that. It’s a sin… That’s what my belief is. I don’t want to do that… I take photos of [my baby with CCHD who passed away]*,* and she’s a part of my kids.”* – Pasifika mother (P16).

*“The thing is is that we’re [the medical team] just gonna give you palliative care.’ I’m like*,* ‘What is that?’ She’s like*,* ‘We’re [the medical team] just gonna care for you and baby for as long as we can for when baby is born.’ Then*,* I said*,* ‘Well*,* that’s really strange because the doctor in Auckland said that her heart was operable. Now you’re telling me that it’s not.”* - Māori mother (P2).

*“Initially*,* they [the medical team] told me to terminate him because he will go through so many things*,* but me and my husband*,* we were not ready to do it because we will not kill someone who is already inside.”* - Asian mother (P12).

Some healthcare professionals reiterated these cultural predicaments on their journeys to increased cultural awareness.

*“I think we [the medical team] should be culturally aware for everyone.”* – Doctor (HCP12).

*“I’m becoming more and more aware of privilege and racism issues and all those things that are inextricably bound up with my life and the health system and my experience of treating patients.”* – Doctor (HCP14).

*“I think that acknowledging and respecting other cultures*,* other methods of doing things*,* other healthcare models*,* is really important because Western medicine is definitely not perfect.”* – Doctor (HCP17).

Lastly, some parents clarified which areas of healthcare expectations were unmet. Some minoritized parents expressed a need for a more humanized and respectful approach to healthcare delivery. One mother (P4), with English as a second language and mental health challenges who lived in a poor rural area, stated,

*“I think that especially with pregnancy and women*,* miscarriages and all that*,* we [society and medicine] forget that we [mothers] have feelings and emotions… we’re humans dealing with humans*,* not humans dealing with cars”.*

Another mother, of Pasifika descent (P19), felt:

“I’m not good enough, or maybe they [the medical team] don’t want to help my baby.”

With an Asian mother (P12) expressing,

*“I’m a human*,* and they [the doctors] didn’t consider me as human… the doctors should be more sensitive”.*

### Category two: Healthcare systems are under-resourced to provide equitable support for the differential needs of grieving parents

All parents recounted grief following the CCHD diagnosis.

*“What grief do you want? Pick your grief. Terminate the child now regret it for the rest of your life. The what if. Decide not to do the surgery*,* watch your baby suffer and die [crying*].” - Māori mother (P5).

Grief and trauma were also apparent for mothers carrying the child, particularly in relation to those with life-limiting palliative conditions.

*“I think for me*,* it’s really hard cos I’m the one carrying him [fetus with life-limiting condition].”* - Pasifika mother (P19).

Trauma was commonly described by parents, one calling her experience:

*“Absolutely horrific… I never really understood PTSD [post-traumatic stress disorder] until I hear that noise [of the hospital monitors].” - European mother (P15)*.

All parents identified a need for wide-ranging support (financial, emotional, cultural, psychological). However, systems and supports did not appear to encompass all parental needs and usually were delivered through charities. For example:

*“I think just checking up more [after neonatal death] … sometimes other [non-family] support is better because sometimes you cannot talk to your own whānau [family]. Sometimes it can be a taboo situation [losing a child]*,* and people just don’t wanna talk about it.”* – Māori mother (P2).

*“I just don’t get any time to just breathe; it’s just still a little bit difficult… The guilt was horrible… and*,* ideally*,* we would have his dad here*,* but one for his mental health*,* he needs to be at work because he feels like that’s the only way he’s being productive and helpful is by making money and paying the bills.”* - Māori mother (P21).

The health professionals acknowledged the difficult situation parents are in when a CCHD diagnosis occurs, with some expressing exasperation at the systemic constraints when supporting non-European families. Support for families was identified by some health professionals as being sub-optimal for different family needs and not always accessible equally to all families.

*“I think traditionally it’s been white middle-class people who have resources that access things like Heart Kids [charity and peer-support group].”* - Midwife/nurse (HCP10).

Non-European parents mentioned the role of communities, spirituality, religion, family, and culture in assisting coping and decision-making. For instance, Te ao Māori practices (Māori language, customs, and protocols) were turned to by Indigenous Māori parents while navigating through the CCHD diagnosis and management, using karakia (prayer) and family support, which was viewed as an area that healthcare systems and staff could support more. Excerpts supporting the broader role of cultural support include:

*“That’s the thing. I was trying to call them [mental health support services]*,* but it took a bit too long. I needed someone right then*,* so I ended up going to just an aunty.”* - Māori mother (P14).

*“They let me give my taonga [treasured cultural possession] and put it in a bag and put by baby’s bed so that he took that with him into surgery.”* - Māori mother (P5).

“*When my son was born*,* my Dad wanted to come into NICU to do karakia [prayer] and he was denied because they [the medical team] said that might offend other families that were around.”* - Māori mother (P21).

The need to incorporate more Indigenized systems was identified by a Māori mother (P21): “I think it just needs to be opened up a lot more. I think, too, more accepting of like maybe te ao Māori practices and understandings” and one doctor (HCP17) agreed, “Māori understandings of life that is a little bit different to Western culture and Western concepts, but equally valid and actually probably support more holistic understanding than we practice in Western medicine”; with participant beliefs being that the current prevailing health practices delivered by a predominantly “White team” **(**midwife/nurse, HCP4) limit the support available for those belonging to less represented groups. Differential support needs were also evident for parents from Asian and Pasifika backgrounds:

*“I was talking to my husband*,* saying*,* ‘I think he’s [their terminated fetus] a fish from the past life… That made me feel peaceful.”* - Asian mother (P18).

*“We had a prayer… And then we came straight from there [hospital] to the funeral home… It was a tough time cos we didn’t work like we didn’t have enough money to cover all the costs*,* but his aunty help us pay all the stuff [for their child’s funeral].”* - Pasifika mother (P19).

In turn, some healthcare professionals quoted a lack of resources, acceptance and understanding as reasons why some parents of minoritized ethnic groups experience sub-optimal support while grieving and adapting to a CCHD diagnosis. Communicating frustration for the constraints of the system they are restricted to work within:

*“Our lack of understanding of what’s important culturally for these [minoritized] families going through this [CCHD] journey and not having the right information and resources available to them.”* - Doctor (HCP6).

*“[We are] doing very well on very less*,* due to the goodwill of the people everywhere*,* because they’re [the medical team] not just doing 100%*,* they’re doing 110% of what they’re meant to do*,* just because of the [limited] resources.”* – Doctor (HCP11).

*“We have our own resource limitations*,* can’t always see them straight away [in clinic]. So*,* I would say that’s some of the system barriers.”* – Doctor (HCP20).

### Category three: Healthcare systems could engage minoritized families more optimally in shared decision making

Healthcare practitioners identified partnership with parents as a priority when delivering healthcare. However, healthcare systems were found to inadequately educate, empower and partner with some Indigenous and minoritized families compared to their European counterparts. Healthcare participants note a lack of educational resources, system barriers and cultural differences when providing healthcare. For instance, one doctor found that European parents may be more directive in communicating their wants and needs for their child. For example:

*“The Pakeha [European] children*,* in many cases*,* the parents are overly anxious and overly worried*,* and get the child pushed ahead [for cardiac surgery].”* – Doctor (HCP16).

In contrast, some healthcare providers viewed their role within a bidirectional partnership to inform and support families respectfully with their unique CCHD management choices. Some examples of how healthcare professionals viewed the patient-provider partnership were:

*“Say the technology’s [available] there*,* the option’s there*,* and say we as a partnership [with the family].”* - Doctor (HCP7).

*“It’s not my decision. It’s you that has to live with whatever you choose”* – Doctor (HCP20).

*“We are here to help you find the best path for you*,* so it’s okay. I’m not going to judge you on that.’”* – Doctor (HCP11).

Rather than disease-focused, an evolution was perceived in how patients are now central to their care and actively involved with the healthcare professional in shared decision-making.

*“[previously*,* we would] talk about what was wrong with her baby. So*,* all of that time*,* she’d had no conversational understanding. She’d had a series of different people who were very interested in imaging her baby’s heart*,* but not necessarily engaging with her over the process and the journey she was undertaking.”* – Doctor (HCP15).

*“Now… it’s more and more of a negotiation with families.”* – Midwife/nurse (HCP19).

The way the initial abnormality was detected in the fetus also affected how parents navigated the experience, as some were unaware that anatomy screening was intended for more than determining the sex of the fetus. Examples of poor experiences from inadequate pre-conception or pre-screening education for Māori and Pasifika mothers included:

*“I never knew there was such things as babies being born with heart conditions.”* - Māori mother (P14).

*“They were just very … I don’t know. The obstetrician didn’t really give me much information and they were just staring at me. It was very awkward. It was a very weird time.”* - Māori mother (P5).

*“The baby’s heart*,* the doctor say about baby’s heart. I don’t know what they say.”* - Pasifika mother (P7).

In addition, educational challenges from resource limits and inequitable systems were perceived by healthcare professionals as contributory to differential parent experiences and understanding levels, as indicated below:

*“Well*,* lots of our resources aren’t in different languages. That’s a bit of a barrier.”* – Midwife/nurse (HCP9).

*“I am really concerned about the lack of equity across the cultures*,* even within our own service… without realizing it*,* we are letting Māori children in particular*,* and some Pacific children*,* go way beyond their [surgery] dates.”* – Doctor (HCP16).

Furthermore, the translation of the healthcare professional-patient partnership was suboptimal for some parents. In part, this was influenced by how parents were enabled to engage actively with the healthcare system, which varied by ethnicity. For example, European parents’ entitled positioning privileged them to participate in decisions and advocate on behalf of their child:

*“So*,* ‘I don’t want to be a pushy hypochondriac and demand that I’m entitled to all these different tests*,* but this is what I’ve been told*,* and I know that this is the case.’”* - European mother (P15).

*“I guess if you wanted more*,* you’d have to advocate for yourself*,* you know*,* it comes back to that: Are you strong enough to advocate for your needs?”-* European mother (P10).

Comparatively, Māori, Pasifika and Asian parents reported less accessibility to participate in decisions and have their needs met.

*“Because of the complex situation*,* [the doctors told us] we’re not gonna do ventilator stuff… the first question he asked me when I told him [his brother] she passed away*,* he said*,* ‘Why didn’t the doctors put her on the vent [ventilator] and try to save her?’”* - Asian father (P1).

*“I think they [the medical team] try to [listen to my requests]*,* but for them*,* it’s a lot like science-based*,* doctor based and then the family come afterwards*,* what the mother thinks.”* - Māori mother (P21).

## Discussion

We present results from 57 interviews of 45 health professionals and parent’ experiences of CCHD in NZ. Our findings of experiences of inequitable quality of healthcare by ethnicity are consistent with a recent paper describing a potential link between disparate access to high quality healthcare and differential mortality by race, [25] and could explain, in part, the previously reported disparate mortality for HLHS infants from Māori and Pacific families in NZ [10, 11, 24]. 

The three lessons learned from the study participants’ narratives are explained in context of the current literature with recommendations presented on future interventions and research directions which could improve equity. These findings also add to the understanding of healthcare professionals’ delivery of fetal care, [26] and their engagement in reducing healthcare disparities, for which information is sparse [27]. Our findings also validate previous reports on quality of hospital experiences for Māori and Pacific peoples within the NZ mainstream healthcare system, which were reported to be unconducive to meeting their needs [28, 29]. 

We identified that minoritized groups experience disparate healthcare quality, which is a timely contribution when equity in pediatric critical care is an international priority [9, 30]. Parents from marginalised groups voiced that they were treated differently by healthcare professionals and were not offered the same care as European parents. Our findings build upon previous research in other settings identifying gaps in quality of healthcare for patients of minoritized ethnic and racial groups [28, 31, 32]. Systemic racism has been suggested as an underlying mechanism in differential care quality, [32–36] supporting a recent American Heart Association call to action to name racism and focus interventions accordingly to reduce disparities in CHD [37, 38]. 

Underlying implicit biases, [32–36] unconscious negative attitudes or prejudices based on stereotypes that all people possess, [39] are likely compounded by structural racism, which may explain outcomes in this study [25, 40–42]. Braveman defined structural racism as –

“forms of racism that are pervasively and deeply embedded in systems, laws, written or unwritten policies, and entrenched practices and beliefs that produce, condone, and perpetuate widespread unfair treatment and oppression of people of color, with adverse health consequences.” [33].

Quality of care may be improved if there is patient-physician racial concordance, clearly not the case in this study as demonstrated by the demographics of the patient and physician groups [45, 46]. Patient-informed experiences could inform healthcare quality in areas of communication, education, support and shared decision making [43, 44]. Improving healthcare quality and equity through mitigating disparities in CHD informed by the insights of underrepresented groups is suggested in a recent American Heart Association statement [37]. 

We found healthcare systems are under-resourced to provide equitable support for the differential needs of grieving parents. We also found grief, psychological distress and post-traumatic stress in families diagnosed with CHD (critical and non-critical); including experiences of families who elected to terminate the pregnancy is a strength of this study and an addition to the literature [4, 12, 47–50]. Our data support the contention that presenting families facing perinatal death with culturally-informed coping strategies could be of benefit [51]. We therefore propose respecting and including holistic support practices, which may include prayer, cultural rituals, community involvement or religious activities, particularly at increased times of need after a critical congenital diagnosis, neonatal death, or termination, where appropriate. A critical finding is that healthcare providers are aware of the multilevel barriers to equitable healthcare delivery and support for CCHD families in NZ, a prerequisite if measures to address these barriers are to be successful.

We report that healthcare systems could engage minoritized families more optimally in shared decision-making. Despite partnership in shared decision-making being a priority voiced by healthcare professionals, the translation of this principle was mixed. The limited experiences of shared decision-making expressed by parents of minoritized ethnic groups addresses the gap identified by Perez Jolles’ systematic review, which found inadequate representation of minoritized populations in pediatric hospital-level research about shared decision-making [44]. A potential contributor to differential experiences of shared decision-making mentioned by some healthcare professionals in this study could be the difficulty gaining rapport with some families, impacting the implementation of partnership and culturally safe healthcare even if this is identified as a need [43, 44]. 

The strengths of this study are the inclusion of parents’ and health professionals’ experiences of CCHD throughout NZ, inclusion of parents from multiple ethnic backgrounds and situations who are under-represented in qualitative CCHD research, [12] and the fact that we conducted two interviews with parents, first during the acute diagnostic period and then during the management period, which reduces recall bias and provides a rich data set of patient experiences. Although results may be limited in their generalisability because of the setting in a country with particular characteristics, they are potentially transferable across Indigenous and minoritized populations and multi-ethnic settings beyond New Zealand.

Potential bias can occur in all qualitative research; however, this was mitigated through continuous discussion with cultural oversight and co-author accountability. Moreover, a rigorous approach was undertaken using the COREQ checklist, [23] member-checking and reflection. Furthermore, as some chose not to be involved in this study, selection bias could have occurred. It is acknowledged that increasing the number of data coders may have enhanced dependency of results.

Our findings offer implications for policy, practice and future research. For policymakers, this study brings to light the need to collaborate with Indigenous and minoritized groups to inform policy, from developing quality indicators and evaluation procedures to implementing recommendations into practice. Moreover, adjusting policies informed through disciplines outside of CHD to mitigate the negative impact of social determinants of health on survival is critical, as suggested by Cherestal and colleagues [52]. The potential for co-governance and unconscious bias training for healthcare professionals to modify the observed inequities could also be considered [39]. 

At the institutional level, study results present future targets for equity interventions in healthcare delivery to enhance patient experiences. For instance, prioritizing delivery of culturally-appropriate, sensitive, high-quality healthcare monitored by clinical audit and reported back to clinical teams is needed to optimize wellbeing during challenging psychological clinical situations for minoritized groups (Indigenous, non-Europeans, LGBTQ+, residents from high poverty rural areas) [51, 53]. An improvement in clinical care and access for pregnant people from minoritized ethnic and racial groups, including those with fewer economic resources, may improve outcomes in CCHD [42]. 

At the healthcare professional level, due to the immense trauma and grief associated with CCHD, there may be a role for humanising parents through applying trauma-informed healthcare practices [54, 55]. We suggest relaying to families that we see you, hear you, and value and respect all of you and your heritage. To this end, adequate time, physical space, and cultural training for staff are required to ensure that all families feel empowered to partner in decision-making, free from shame or stigma. Moreover, there is space to strengthen the multidisciplinary roles within clinical medicine to more holistically support patients and families with CCHD and infer more time to clinicians. Diversifying the charity organisation support, information sources and peer support to better reflect the population healthcare units service could be a practical next step in enhancing parent experiences in CCHD.

Diversification of the backgrounds of multidisciplinary healthcare staff and doctors through supportive CHD training pathways is of theoretical benefit and endorsed by a recent statement from the American Heart Association [37]. Training of healthcare professionals in cultural safety to enhance trust, confidence, and rapport with minoritized ethnic groups is required to optimally transition minoritized groups from passivity to more active shared decision-making [43, 44]. Likewise, in-service hospital role modelling of non-biased, high-quality care to minoritized population groups needs to occur, alongside contemporizing medical undergraduate teaching curricula to reflect the needs of all patients. In this way, a multilevel, longitudinal approach to healthcare professional development can be targeted which could improve equity for ethnic groups most at risk of poor experiences and health outcomes.

Future mixed-method inquiries and interventional research applying and assessing successful equity strategies in CCHD are needed; these also could address whether variations in healthcare quality and shared decision-making by parent ethnicity are associated with mortality and morbidity outcomes. Research in other jurisdictions would help confirm our findings from the NZ setting. Clarifying patient preferences and unique needs during pregnancy termination and child loss is a necessary next step to inform the development of sensitive processes and systems in this challenging area [53, 55]. Moreover, further defining and monitoring associated risk-factors for mortality by ethnicity in CCHD would be of benefit. More challenging is research into the role of implicit bias may play in determining outcome and the utility of mandated training in this area [39], and whether ethnicity of the healthcare professional contributes to variability in counselling practices.

## Conclusion

According to the experiences of parents and healthcare professionals, persisting inequities in quality of CCHD healthcare (communication, education, support and shared decision-making) occur by ethnic group, with the NZ healthcare system privileging European families. Healthcare leaders, policymakers, and professionals could apply the information identified to construct a healthcare system that is more fit for purpose for all. Further qualitative studies and interventional research into advancing health equity is suggested.

### Electronic supplementary material

Below is the link to the electronic supplementary material.

Supplementary Material 1: Table 1: Kaupapa and Talanoa principles and their application in this research. Table 2: Healthcare professional semi-structured interview questions and prompts. Table 3: First semi-structured interview questions and prompts for parents. Table 4: Follow-up interview questions and prompts for parents. Table 5: COREQ 32 item checklist. Table 6: Categories, subcategories and quotes.

## Data Availability

The datasets generated and/or analysed during the current study are not publicly available to retain participant confidentiality but are available from the corresponding author on reasonable request in a deidentified format. All requests will be reviewed by the Liggins Institute Data Access Committee.

## Abbreviations

CHD: Congenital heart disease
CCHD: Critical congenital heart disease
HLHS: Hypoplastic left heart syndrome
MFM: Maternal fetal medicine
NZ: New Zealand
