# Supplementary material for: Parent and healthcare professional experiences of critical congenital heart disease in New Zealand to advance health equity
Source: BMC Health Serv Res. 2024 Aug 26;24:991. doi: 10.1186/s12913-024-11410-4 (PMC11348529; doi:10.1186/s12913-024-11410-4)
Supplement: Supplementary file 1 — Supplementary Material 1: Table 1: Kaupapa and Talanoa principles and their application in this research. Table 2: Healthcare professional semi-structured interview questions and prompts. Table 3: First semi-structured interview questions and prompts for parents. Table 4: Follow-up interview questions and prompts for parents. Table 5: COREQ 32 item checklist. Table 6: Categories, subcategories and quotes. [file 12913_2024_11410_MOESM1_ESM.pdf]

Supplementary materials

Contents

Table 1: Kaupapa and Talanoa principles and their application in this research Page 2

Table 2: Healthcare professional semi-structured interview questions and prompts Page 3

Table 3: First semi-structured interview questions and prompts for parents Page 4

Table 4: Follow-up interview questions and prompts for parents Page 4

Table 5: COREQ 32 item checklist Page 5

Table 6: Categories, subcategories and quotes Page 6

Figure 1: Coding tree Page 11

Table 1: Kaupapa and Talanoa principles and their application in this research

Kaupapa Māori principles	Talanoa principles	Description	Application in research method
Āta<br><br>Establishing respectful relationships	Relationships/Alofa*	Relationships are to be developed slowly and deeply with intention, love and respect.	Nurturing and respecting the families involved in the interview study and acknowledging their strength, courage and determinationalongside their vulnerability; this was practically outworked through ethnically derived recruitment and using a Māori interviewer for Māori parents. Engagement was meaningful through offering parents to meet in person and at a place of their choosing.
He taonga tuku iho<br><br>Nurturing and caring for the treasures handed down from their ancestors	Respect/Fa'aaloalo* derived from the root word 'alo' (face-to-face)	Māori Tikanga (customs) and Pacific customs are treasured, and native language is respected in relationships between the researchers and those being researched.	Consent forms were offered in six languages and interpreters offered for all of the interviews. Cultural customs were also always offered such as koha or meaalofa* (gift), karakia (prayer) and kai (food) and purposefully incorporated into interviews. Meetings were offered face to face and interviewers travelled around the country to accomodate in-person interview requests. For example, meeting in person allowed for more significant interactions such as the whānau (family) sharing photos, talking about baby in the present, drawing on cultural myths and legends determining meaning in the experience.
Ako<br><br>Affectionately and authentically developing connection	Meaningful engagement	Promotes reciprocity to teach and to learn. The relationship and connection built with Māori and Pacific people are founded on a mutual balance and partnership.	When analyzing the dialogue in the transcripts from interviews the parents' genuine lived experiences were appreciated and upheld through viewing them as the 'authority' and 'expert' during the research process. Parents also lead the dialogue through the semi-structured interview approach. Development of a deeper connection which allowed for a more meaningful engagement with parents was possible due to interviews with parents being offering at two different time points.

(Table adapted from Brown, 2018; Cram & Adcock, 2024) *Samoan equivalent

Table 2: Healthcare professional semi-structured interview questions and prompts (final version)

Offer Karakia [prayer] where appropriate.<br>Hui Process Mihimihi/ Whakawhānaungatanga (introductions and greetings).<br>Verbally re-confirm consent.	
**Key questions**	**Prompts***
Where are you from and what nationality do you identify with?	• *Where did you grow up and undertake healthcare training?*<br>• *What is your background?*
Please tell me about your current clinical role	• *What are the best and hardest parts of your role?*<br>• *What do you see as the role of a doctor/midwife/nurse…*
What is it like working with CCDH families?	• *Tell me about a time this went well, didn't go well.*<br>• *What do you think is bad news?*<br>• *What do you think about when you know you have to give bad news?*
How do you approach disclosing diagnoses and counselling families with CCHD?	• *Do you adapt your approach based on the characteristics of families?*<br>• *How do you perceive your role in the patient-provider relationship?*<br>• *Do you have any relevant examples to illustrate this?*
What influences family coping with a CCHD diagnosis?	• *Why do you say that?*
What influences your decision-making when managing CCHD babies and their family?	• *Do you adapt your approach based on the characteristics of families?*<br>• *Do you have any relevant examples to illustrate this? Could you comment on how power and privilege may play a role?*<br>• *Do you agree with the patient partnership model and why?*
What patients make you pause to consider how best to approach a family interaction?	• *Why do you think this is?*<br>• *How do you perceive and manage any potential assumptive narratives or stereotypes of your patients you may encounter?*<br>• *Which characteristic traits pose greater challenges?* *I.e., ethnic groups, or age, or numbers of people in a room.*
Which groups have issues accessing and engaging in health services?	• *i.e., Which groups are difficult to contact, follow-up and miss appointments?*<br>• *Do you have any relevant examples to illustrate this?*<br>• *Why do you think this?*
We know there are ethnic disparity in health outcomes in CCHD in NZ. What are your thoughts about why that is?	• *Why do you say that?*<br>• *Do you have any relevant examples to illustrate this?*
How do you think we can improve equity of outcomes and the parental experience of CCHD in NZ?	• *If you had endless resources to improve the current health care system, accessibility and model of clinical care what would you do to improve equity?*
Did knowing about this study change your clinical practice and how?	• *What have you noticed in response to your change in practice?*
Is there anything I haven't asked you that you want me to know?<br>Koha [voucher] and thank you	

**Note: Non-leading and general prompts may also be used.*

Table 3: First semi-structured interview questions and prompts for parents (final version)

Offer Karakia [prayer] where appropriate.<br>*Hui Process Mihimihi/ Whakawhānaungatanga (introductions and greetings).*<br>*Verbally re-confirm consent.*
To begin, can you please tell me about yourself and your family/whānau and where you come from?
What does your ancestry / heritage mean to you?
How has the pregnancy been?
How did you come to find out about baby's heart?
What was it like to be told about baby's heart condition?
What thoughts and feelings have you had since finding out about baby's heart?
How do you describe what has happened to your baby to others?
How has hearing the news about baby's heart impacted you and your family/whānau?
What has assisted or hindered your adjustment to this unexpected news?
Where you offered support through your journey and what were they? *[Spiritual/ cultural/ emotional/ financial/ other]*
How much input have you had into deciding what is happening with your baby?
Do you wish anything had gone differently about how you learned about your baby's heart problem? *If so, what would you change and why?*
Is there anything I haven't asked you that you want me to know?
Interview end. Thank you.<br>*Whānau/family given koha [voucher] and supports offered to everyone.*

Table 4: Follow-up interview questions and prompts for parents (final version)

Offer Karakia [prayer] where appropriate.<br>*Hui Process Mihimihi/ Whakawhānaungatanga (introductions and greetings).*
How have things been going since I last saw you?
Have there been any changes in how you understand your baby's heart?
What has your experience been with health services and systems? (For example, communication, clinics, hospital, cultural/spiritual/general support offered)
What has influenced your decisions around care for your baby?
What is your understanding and opinion on the care for your child?
How do you feel about access to health services in general?
What do you value from an interaction with health services?
What are your thoughts on equality of treatment from health providers and our health system in general in Aotearoa/New Zealand?
Is there anything you wish was different about care has been communicated and given for your child?
Is there anything I haven't asked you that you want me to know?
Interview end. Thank you.<br>*Whānau/family given koha [voucher] and supports offered to everyone.*

Table 5: COREQ 32 item checklist

**Domain 1: Research team and reflexivity**	**Page and line no.**
Who conducted the interviews is reported clearly	Page 7, lines 75-77
Credentials of interviewers	Page 7, lines 75-77
Occupation of interviewers	Page 7, lines 75-77
Gender of interviewers	Page 7, lines 75-77
Experience or training the researcher had described	Page 7, lines 75-77
No relationship with participants was established prior to study commencement	Page 6, lines 63-64
No participant knowledge of researchers conducting consent process and interviews	Page 6, lines 63-64
Personal characteristics of interviewers reported	Page 7, lines 75-77
**Domain 2: study design**	
Methodological orientation and theory identifiable	Page 5, lines 26-31
Sampling of participants	Page 6, lines 44-56
Method of participant approach	Page 6, lines 44-56
Sample size	Figure 1
Non-participation reported	Figure 1
Setting of data collection	Page 5, line 27
Presence of non-participants	Page 8, line 93
Description of sample demographic data	Table 1
Interview guide	Supplementary tables 2-4
Repeat interviews	Page 4, lines 78-79
Audio/visual recording	Page 7, line 79
Field notes	Page 7, line 85
Duration	Table 1
Data saturation	Page 7, line 86
Transcripts returned	Page 8, line 90
Number of data coders	Page 8, lines 90-93
Description of coding tree	Supplementary figure 1
Derivation of themes	Supplementary table 6
Software	Page 8, line 89
Participant checking	Page 9, line 117
Quotations presented	Throughout results
Data findings consistent	Throughout results
Clarity of major and minor themes	Supplementary table 6

(Table adapted from Tonga, 2007)

Table 6: Categories, subcategories and quotes

Category (subcategory)	Healthcare professional quotes	Parent quotes
Minoritized groups experience disparate healthcare quality<br><br>(Communication, stereotyping and bias differ)	"Māori understandings of life that is a little bit different to Western culture and Western concepts, but equally valid and actually probably support more holistic understanding than we practice in Western medicine." – Doctor (HCP17)<br><br>"Particularly I would say the Asian, Far Eastern cultures. They want perfection… I think there's a lot of whānau [family] pressure [to terminate] … They [the family] want to have a perfect child and if they haven't got a perfect child, they could be ostracised. That's the suspicion I have from interactions." –Doctor (HCP3)<br><br>"I think there may be biases there where people do make decisions that includes ethnicity. I don't know. I hope not. You'd really hope that we don't do that." – Doctor (HCP14)<br><br>"I think in reality we are not totally aware of our own biases and our own barriers that we put in place that prevent us from providing the best healthcare to everyone where they're at, so unless you actually prioritise it across the board, it is never gonna really happen, and I think people are at different stages of acceptance of that on an individual level." – Doctor (HCP17)	"How Māori look at health is very holistic. How I feel Pākehā [Europeans] tend to look at health is very quite clinical…" - European mother (P22)<br><br>"We're going through something; we need to know everything straight away. No need to hide anything from us." - Asian father (P1)<br><br>"To be honest, I think that they would go the extra mile for — well, it's pretty sad to say, but they would go the extra mile for Pākehā [Europeans]." - Māori mother (P2)<br><br>"Because of the complex situation, [the doctors told us] we're not gonna do ventilator stuff. Well, I don't blame them because they did try their best, but when I talked to my brother overseas, the first question he asked me when I told him she passed away, he said, 'Why didn't the doctors put her on the vent and try to save her?'" - Asian father (P1)<br><br>Thank you for saving our [child's] life because essentially if that [congenital cardiac] service wasn't there, he wouldn't be here. So, we are very lucky in that respect… obviously we have a great health system." - European mother (P11)<br><br>"The whole time we were in NICU, although it's not a place that you want to be, like we were aware that we're really fortunate in New Zealand to have such a level of care." - European mother (P9)<br><br>"To be fair, we were blown away by how good everyone was." - European father (P25)

Minoritized groups experience disparate healthcare quality<br><br>(De-humanisation and acceptability of care experiences)	"I think some of it is some groups are more accepting of the medical model of care and coming to the hospital." – Doctor (HCP2)<br><br>"So rather than just a number, we have to see the patient as a person and what's helpful for them?" – Doctor (HCP7)<br><br>"I think they get labelled as difficult families and frequent non-attenders, but they might have a really, really good reason as to why that's the case." – Doctor (HCP6)	"I know the doctors want to understand, but I don't think they do understand… Don't make assumptions because you don't know." - Māori mother (P21)<br><br>"There's another thing I read was that you get treated like a number until you're actually in Auckland and they can see you." – Māori mother (P6)<br><br>"But then the other thing was just as a concept people using baby's name like they exist. Some people like that, some people don't, that's the thing. But in all of the messages of support, rather than acknowledging us as parents, it's more of the inclusion of baby into the conversation, I guess, was helpful because then it's like that subconscious connection that they [the aborted fetus] existed is the main thing." - European mother (P10)<br><br>"I'm a human and they didn't consider me as human." - Asian mother (P12)
Healthcare systems are under-resourced to provide equitable support for the differential needs of grieving parents<br><br>(Grief and trauma response and the supports available)	"I think every patient could do with a karakia [prayer] to open it." – Doctor (HCP1)<br><br>"I mean, I definitely see when I talk with families postnatally, generally it's your Pākehā families that have joined [HeartKids]." - Midwife/nurse (HCP5)<br><br>"Because we're a pretty white team, particularly with cardiac and the fetal medicine team." – Midwife/nurse (HCP4)<br><br>"Our lack of understanding of what's important culturally for these families going through this journey and not having the right information and resources available to them. But then also part of that is us educating them and they're not being able to understand, from our point of view, the importance of the health care that we're trying to provide for them." - Doctor (HCP6)	Pick your grief. Terminate the child now, regret it for the rest of your life. The what if. Decide not to do the surgery, watch your baby suffer and die [crying]." - Māori mother (P5)<br><br>"I did ask them could we have a karakia, but they said we can't cos of COVID." - Māori mother (P5)<br><br>"Really hard to accept what happened, but the Church that we came from, it teaches us so we can understand and know if something bad happened to the baby. If he passed, we can meet with him on the other side." - Pasifika mother (P19)<br><br>"I think it just needs to be opened up a lot more. I think, too, more accepting of like maybe te ao Māori practices and understandings" - Māori mother (P21)

		"I never knew there was such things as babies being born with heart conditions." - Māori mother (P14)<br><br>"'What's going on?' She [the ultrasonographer] said, 'I'm really sorry. The baby's heart looks very abnormal. You're gonna have to see a specialist" … I was in a bit of a pit of depression for a while, just trying to get my head around it all "- Māori mother (P6)
Healthcare systems are under-resourced to provide equitable support for the differential needs of grieving parents<br><br>(Systemic barriers and resource limits)	"Our environment is not really set up for lots of family to be around if that's what somebody needs… and only having one family member stay overnight and things is challenging for some families. Also, our clinics are quite inflexible and some families need more time to plan and put plans in place and work out how they're gonna get here. Also, we don't have car parking. Families drive around for two hours trying to get a car park, so where we're located is not helpful for families." – Midwife/nurse (HCP9)<br><br>"…unless we know what are the reasons why they're not coming, we can't address them." – Female doctor (HCP16)<br><br>"[We are] doing very well on very less… they're doing 110% of what they're meant to do, just because of the [limited] resources." – Doctor (HCP11)<br><br>"I think that a whole of society approach would be helpful, if there was less poverty in our communities, that would help, if there were no barriers at all to effective early pregnancy care, so women still have to pay to have scans, and that means they don't have them, basically, if cost is an issue for them… Which are entirely beyond the health system's control." – Doctor (HCP18)<br><br>"There are language issues that we haven't been aware of." - Midwife/nurse (HCP5)<br><br>"I wonder sometimes whether in dealing with these, is it easier for, I guess, doctors of the same community to deal with certain minorities?" – Doctor (HCP11)	"Parking [at hospital] was incredibly difficult. We were an hour late [to our clinic appointment] just because of parking." – Māori mother (P14)<br><br>"Yeah, sometimes my parents got busy with their own stuff and sometimes I have to call my midwife and ask them if I can just come in one day [for my hospital appointments] cos there's no one to stay with the kids." – Pasifika Mother (P19)<br><br>"I think the only thing I really, really struggled with was not having any family support over overnight, but that was the rules of the ward. You're only allowed one parent overnight." - Māori mother (P14)

	"We have our own resource limitations, can't always see them straight away. So, I would say that's some of the system barriers." – Doctor (HCP20)	
Healthcare systems could engage minoritized families more optimally in shared decision making<br><br>(Partnership with patients)	"Say the technology's there, the option's there, and say we as a partnership." - Doctor (HCP7)<br><br>"Now… it's more and more of a negotiation with families." – Midwife/nurse (HCP19)<br><br>"I'd like to think we didn't do that [make different clinical management decisions] on ethnicity. I think we probably do it sometimes on a mixture, so of parents influencing us or asking for certain things. I think that may be different in different ethnic groups. I think some people may be more accepting of what we say rather than demanding that we do more." – Doctor (HCP14)	"Everything is about science and having this hospital, they don't think about how if a mother would think… They don't take in, I think they try to, but for them it's a lot like science based, doctor based and then the family come afterwards, what the mother thinks… it's always gotta be based on what everybody else says." - Māori mother (P21)<br><br>"No, no options were given." - Māori mother (P21)<br><br>"But they promised us that they would be there for us when I gave birth to help me with anything about the baby, but they wasn't there… I was so angry cos they didn't do what we was talking about" - Pasifika mother (P19)
Healthcare systems could engage minoritized families more optimally in shared decision making<br><br>(Empowering and educating families)	"Definitely I think health literacy can be something that can really help a family because if they understand why or what's the basis of it, they're much more likely, I believe, to comply" – Midwife/nurse (HCP19)<br><br>"Health literacy, is a very westernized model that we have, and I think that is very detrimental to not only Māori culture but also lots of other cultures, cos I mean New Zealand's a country of immigrants, so everyone has come from somewhere else originally, and I think we lose sight of that, so yeah, I think, our health literacy model is very much westernized and that although puts up barriers to access and knowledge of accessing both the health system but also accessing support networks, all of that thing" – Doctor (HCP17)<br><br>"Our role is to give the family information… then support them… in that choice 100%." – Doctor (HCP18)<br><br>"The Pākehā [European] children, in many cases the parents are overly anxious and overly worried, and get the child pushed ahead [for cardiac surgery]." – Doctor (HCP16)	"The baby's heart, the doctor say about baby's heart. I don't know what they say." - Pasifika mother (P7)<br><br>"They were just very ... I don't know. The obstetrician didn't really give me much information and they were just staring at me. It was very awkward. It was a very weird time, and they were just kinda, 'Yeah, so you'll have to give birth in Auckland at Starship and you'll have surgery." - Māori mother (P5)<br><br>"I went into the fetal medicine room, you obviously go in there — well, I know I did. I went in there and I was prepared, and I was like, Okay, I have all these questions. But then I got overwhelmed with everything they have come up with. I got a little bit overwhelmed, and I forgot all my questions." - Māori mother (P21)<br><br>"Initially, they told me to terminate him because he will go through so many things, but me and my husband, we were not ready to do it because we will not kill someone who is already inside" – Asian mother (P12)

		"I've read that – cos I like reading all the medical journal stuff and I'd read that it's like if she's got a virus, having a general anaesthetic could be quite dangerous." - European mother (P4)<br><br>"I think that some whānau [families] would be really whakamā [embarrassed], really shy. And again, they would look at a doctor and the nurses and that as above them, so they would just let them do whatever they want. Not ask questions, that kind of stuff… I can imagine some families again would be just too shy. And sometimes you don't want to say something's that dumb or ask a silly question." - Māori mother (P5).

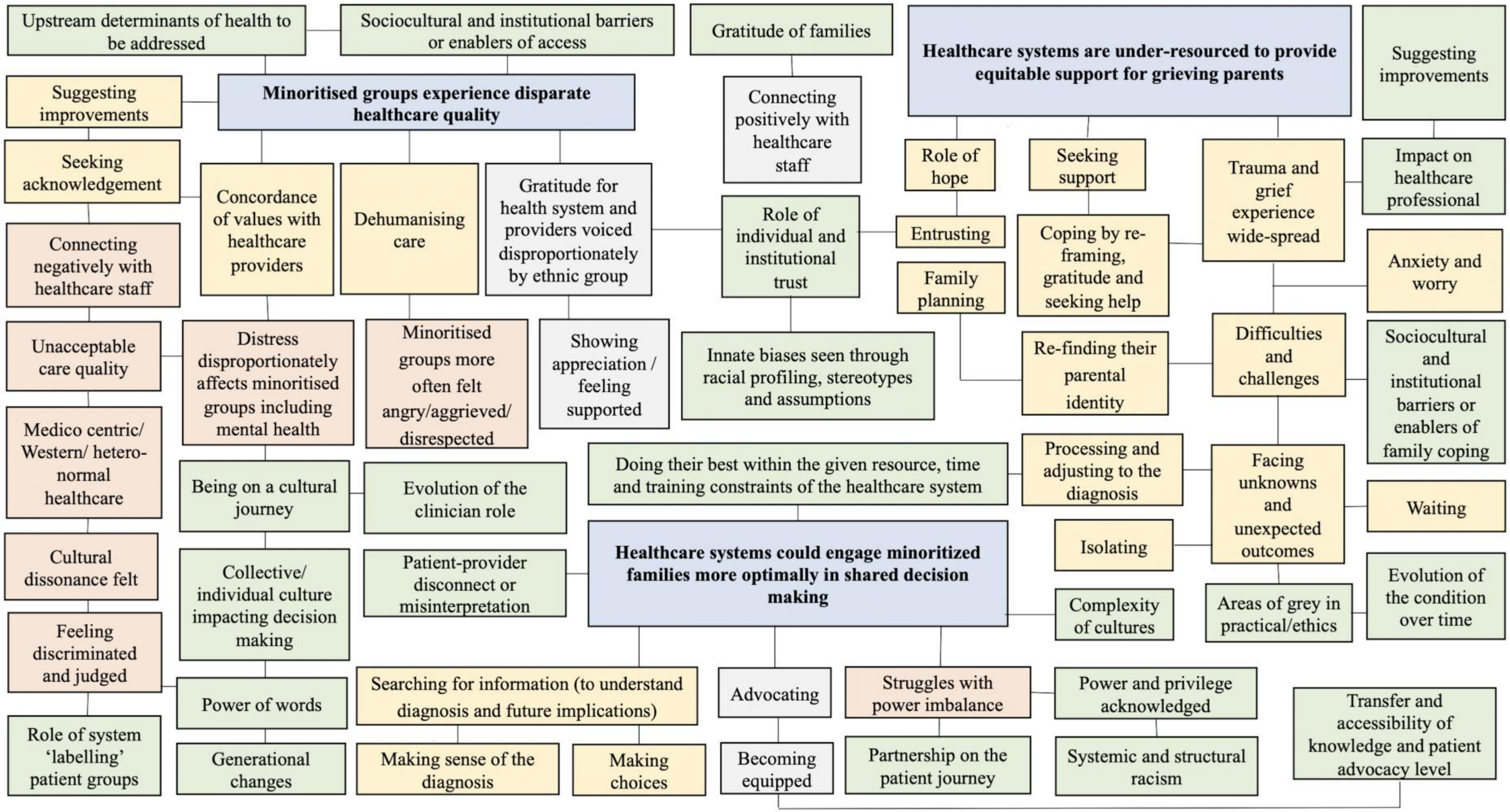

Figure 1: Coding tree

Key: Blue = key categories, green = codes from healthcare professional interviews, red = codes predominantly from Māori/Pacific/Asian parents, grey = majority of codes from European parents, and yellow = common codes from parents.
